# The Association Between Clinical Severity and Incubation Period of SARS-CoV-2 Delta Variants: Retrospective Observational Study

**DOI:** 10.2196/40751

**Published:** 2022-11-18

**Authors:** Kai Wang, Zemin Luan, Zihao Guo, Jinjun Ran, Maozai Tian, Shi Zhao

**Affiliations:** 1 Department of Medical Engineering and Technology Xinjiang Medical University Urumqi China; 2 JC School of Public Health and Primary Care Chinese University of Hong Kong Hong Kong China (Hong Kong); 3 School of Public Health Shanghai Jiao Tong University School of Medicine Shanghai China

**Keywords:** COVID-19, Delta variant, incubation period, clinical severity, China

## Abstract

**Background:**

As of August 25, 2021, Jiangsu province experienced the largest COVID-19 outbreak in eastern China that was seeded by SARS-CoV-2 Delta variants. As one of the key epidemiological parameters characterizing the transmission dynamics of COVID-19, the incubation period plays an essential role in informing public health measures for epidemic control. The incubation period of COVID-19 could vary by different age, sex, disease severity, and study settings. However, the impacts of these factors on the incubation period of Delta variants remains uninvestigated.

**Objective:**

The objective of this study is to characterize the incubation period of the Delta variant using detailed contact tracing data. The effects of age, sex, and disease severity on the incubation period were investigated by multivariate regression analysis and subgroup analysis.

**Methods:**

We extracted contact tracing data of 353 laboratory-confirmed cases of SARS-CoV-2 Delta variants’ infection in Jiangsu province, China, from July to August 2021. The distribution of incubation period of Delta variants was estimated by using likelihood-based approach with adjustment for interval-censored observations. The effects of age, sex, and disease severity on the incubation period were expiated by using multivariate logistic regression model with interval censoring.

**Results:**

The mean incubation period of the Delta variant was estimated at 6.64 days (95% credible interval: 6.27-7.00). We found that female cases and cases with severe symptoms had relatively longer mean incubation periods than male cases and those with nonsevere symptoms, respectively. One-day increase in the incubation period of Delta variants was associated with a weak decrease in the probability of having severe illness with an adjusted odds ratio of 0.88 (95% credible interval: 0.71-1.07).

**Conclusions:**

In this study, the incubation period was found to vary across different levels of sex, age, and disease severity of COVID-19. These findings provide additional information on the incubation period of Delta variants and highlight the importance of continuing surveillance and monitoring of the epidemiological characteristics of emerging SARS-CoV-2 variants as they evolve.

## Introduction

The ongoing COVID-19 pandemic caused by SARS-CoV-2 has been continuously spreading worldwide, posing significant threat and burden to public health systems. The emergence of SARS-CoV-2 variants has accelerated the global spread of COVID-19 [1]. In February 2021, the SARS-CoV-2 Delta variant (Phylogenetic Assignment of Named Global Outbreak lineage: B.1.617.2) was first detected in India [2]. Subsequently, major outbreaks seeded by the Delta variants have been reported in various regions [3,4]. A comprehensive understanding of the epidemiological characteristics of the Delta variant would help inform targeted interventions for containing the spread of COVID-19 [5].

The continuous evolution of new variants of SARS-CoV-2 since the outbreak has been a great challenge, especially for those in health care and research and development in the areas of diagnosis, prevention and treatment development, as well as policy makers and administrators [6], resulting in rapid changes in the epidemiological information used to plan and evaluate strategies to prevent the spread of COVID-19 [7].

The incubation period, defined as the time delay between the onset of infection and symptoms of a case, is an imperative epidemiological parameter of an infectious disease. From the perspective of epidemic control, estimating the incubation period could help determine the quarantine time, develop control measures, and predict the transmission dynamics [8]. Apart from that, the incubation period also plays an important role in determining the proportion of presymptomatic transmission, which has posed significant challenges in the containment of epidemics [9]. Thus, it is of crucial importance to clarify the distribution of the incubation period especially for the SARS-CoV-2 variant, which could cause large outbreaks.

The current understanding on the incubation period for the SARS-CoV-2 Delta variant is limited. Although estimates of the incubation period of various historical SARS-CoV-2 strains can be found in the literature [10-12], knowledge of the incubation period of Delta variants has been largely scarce. However, recent studies conducted in Guangdong province, China, have shown that the Delta variant has a shorter incubation period than non-Delta variants [13,14]. The incubation period of COVID-19 could vary by age, sex, disease severity, and study settings [15]. The impact of these factors on the incubation period for the circulating Delta variant remains uninvestigated.

From July to August 2021, outbreaks seeded by the Delta variant were reported in Nanjing and Yangzhou, Jiangsu province, China, with a larger scale compared to the Delta outbreak that had occurred in Guangdong province from May to June 2021. The aim of this study was to characterize the incubation period of Delta variants using detailed epidemiological contact tracing data collected during the Delta outbreak in Jiangsu. Subgroup analysis was also conducted to examine the effect of age, sex, and disease severity on the incubation period. Furthermore, by applying a multivariate logistic regression model, we investigated the association between disease severity and incubation period of the Delta cases.

## Methods

### Data

Epidemiological contact tracing data of the cases infected with the Delta variant were collected from Nanjing Health Committee of Jiangsu Province [16] and Yangzhou Health Committee [17], from July to August 2021. We extracted the demographic and clinical information for each case, including age, sex, home address, exposure and contact history, date of COVID-19 diagnosis, and clinical severity categorized according to the criteria proposed by the National Health Commission of the People’s Republic of China (ie, asymptomatic, mild, moderate, severe, and critical). Asymptomatic cases and cases that did not have any information on the exposure were excluded when estimating the incubation period.

On July 27, 2021, according to the Nanjing Centers for Disease Control and Prevention, the outbreaks were seeded by the SARS-CoV-2 Delta variants according to the whole genome sequencing results [18]. All cases included in this study were laboratory confirmed through real-time reverse transcription polymerase chain reaction or antigen test on a nasopharyngeal swab. The incubation period is the time delay between the date of infection and the date of onset of symptoms. A transmission pair was identified if 2 confirmed cases had a clear epidemiological link (clearly identified who is infected by whom through the contact history in the dataset which was confirmed by the official published epidemic reports). The date of infection is identified based on the contact history between each infected-infector transmission pair in the officially reported epidemiological survey reports. The time of symptom onset date is identified based on the time of symptom onset for each infected person in the officially reported epidemiological investigation reports. For cases without information on the exact date of infection, exposure windows (with lower and upper bound for the exact exposure date) were determined according to the trajectory and duration of contact.

### Incubation Period

We assumed the incubation period *T* of the Delta cases was a random variable following a gamma distribution. For case *i* with known date of infection *E* and symptom onset *S*, the likelihood function was given by the following:



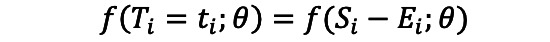



Here, *f* (.) is the probability density function of gamma distribution with parameters denoted by *θ*. For cases identified with an exposure window (*E_Li_*, *E_Ri_*), the incubation period was therefore interval-censored and bounded by (*T_Li_, T_Ri_*)=(*S_i_* – *E_Ri_, S_i_* – *E_Li_*). The total likelihood function was thus formulated as follows:



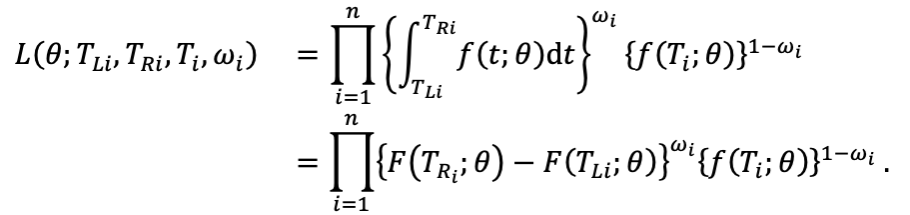



Here, *F* (.) represents the cumulative distribution function, and **ω*_i_*** represents indicator variable. We have **ω*_i_* =** 1 if the incubation period was interval-censored and **ω*_i_* =** 0 if the exact incubation period was observed. The parameters were estimated by Markov chain Monte Carlo (MCMC) method with uniform prior distribution *U(0,100)*. Marginal posterior distributions were obtained from 10,000 iterations, among which the first 5000 iterations were discarded as burn-in period. The 95% credible interval (CrI) was obtained from marginal posterior distributions. We estimated the incubation period distribution for overall cases and for different stratification of cases including age groups (ie, 0-18 years, 19-39 years, 40-59 years, 60-79 years, and over 80 years), sex, clinical severity, and geographical regions (ie, Nanjing and Yangzhou).

### Logistic Regression

Multivariate logistic regression model was applied to examine the associations between the incubation period and disease severity 
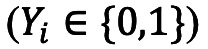
 of the cases infected with the Delta variant. The independent variables including age (*A*), sex (*S*), and incubation period (*T*) were included in the model. For case *i* with known date of infection, the probability *P* that the case's symptom is severe and critical (*Y_i_* = 1) is:



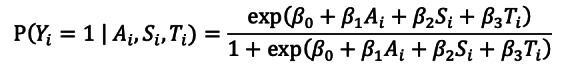



For cases that had a window of exposure, the probability *P* is given by:



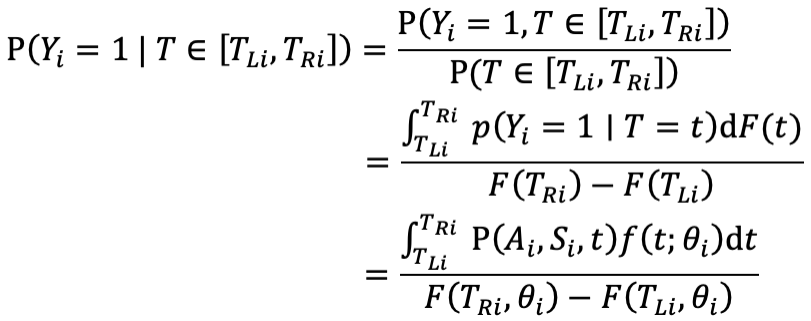



Moreover, we define 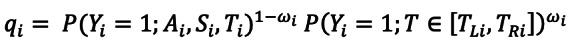. Therefore, the likelihood function was constructed as:



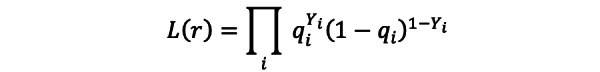



We estimated the coefficients’ vector 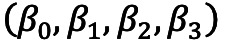 by MCMC with normal prior distribution. The marginal posterior distributions of parameters were obtained from 100,000 iterations, among which the first 50,000 iterations were discarded for the burn-in period. The 95% CrI was obtained from the marginal posterior distributions of unknown parameters.

### Ethical Considerations

The collection of specimens as well as epidemiological and clinical data for SARS-CoV-2–infected individuals and their close contacts were a part of a continuing public health investigation of COVID-19 outbreaks, ruled in the Protocol on the Prevention and Control of COVID-19 by the National Health Commission of the People’s Republic of China, which was exempt from ethical approval (ie, institutional review board assessment). All data used in this study were collected via public domains without personal identity; thus, institutional ethics review was waived.

## Results

A total of 763 COVID-19 cases infected by the Delta variant were reported in Nanjing and Yangzhou from July to August 2021. Of the 763 cases, 410 (53.7%) were excluded due to a lack of exposure history, and the remaining 353 (46.3%) were included in the analysis. Of the 353 included cases, 161 (45.6%) were from Nanjing and 192 (54.4%) were from Yangzhou. In this study, the included cases were divided into two subgroups according to the severity of the disease: age group and sex. The age groups were divided into 5 groups (0-18 years, 19-39 years, 40-59 years, 60-79 years, and over 80 years).

A total of 132 (37.4%) cases aged 40-59 years accounted for a higher proportion than other age groups, with a smaller proportion of children (n=47, 13.3%) and people older than 80 years (n=7, 2%). The proportion of female (n=220, 62.3%) cases was higher than that of male cases (n=133, 37.7%) (Table 1).

**Figure 1:**
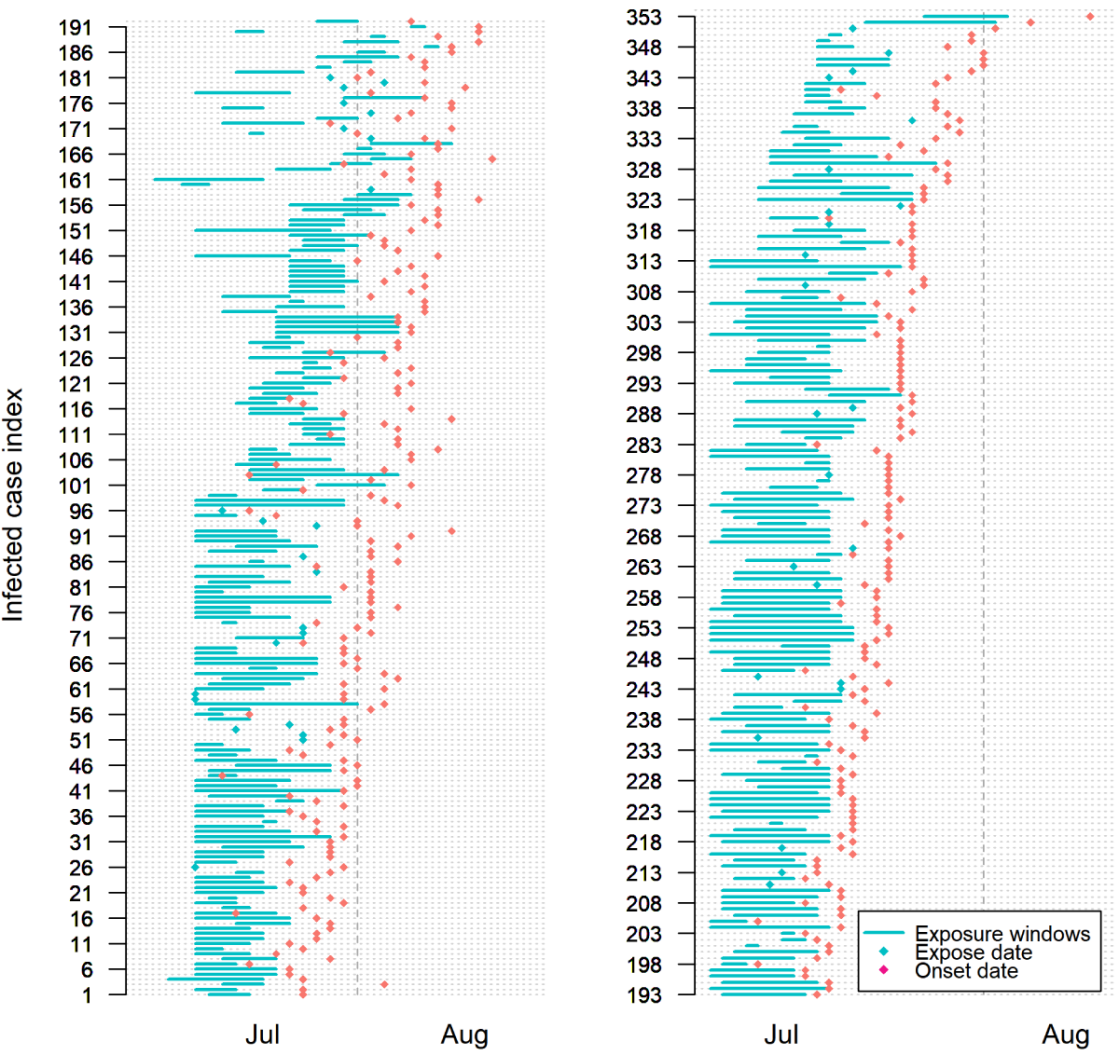
Timeline of the course of infection for each case infected by SARS-CoV-2 Delta variants (n=353) from July to August 2021, in Jiangsu province, China.

**Figure 2:**
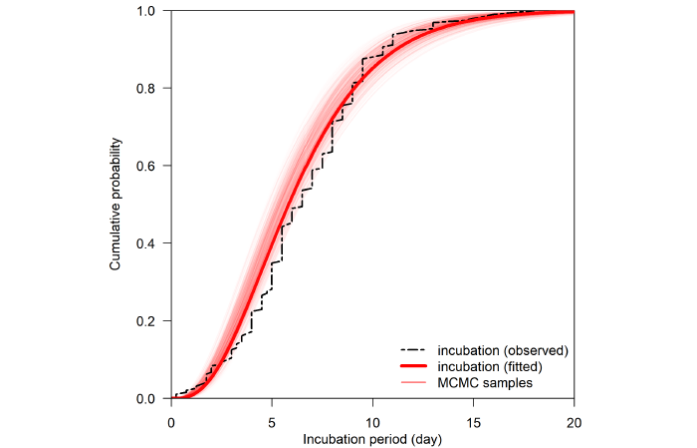
Cumulative distribution of the estimated gamma incubation period for the confirmed SARS- CoV-2 Delta cases (n=353). MCMC: Markov chain Monte Carlo.

**Figure 3:**
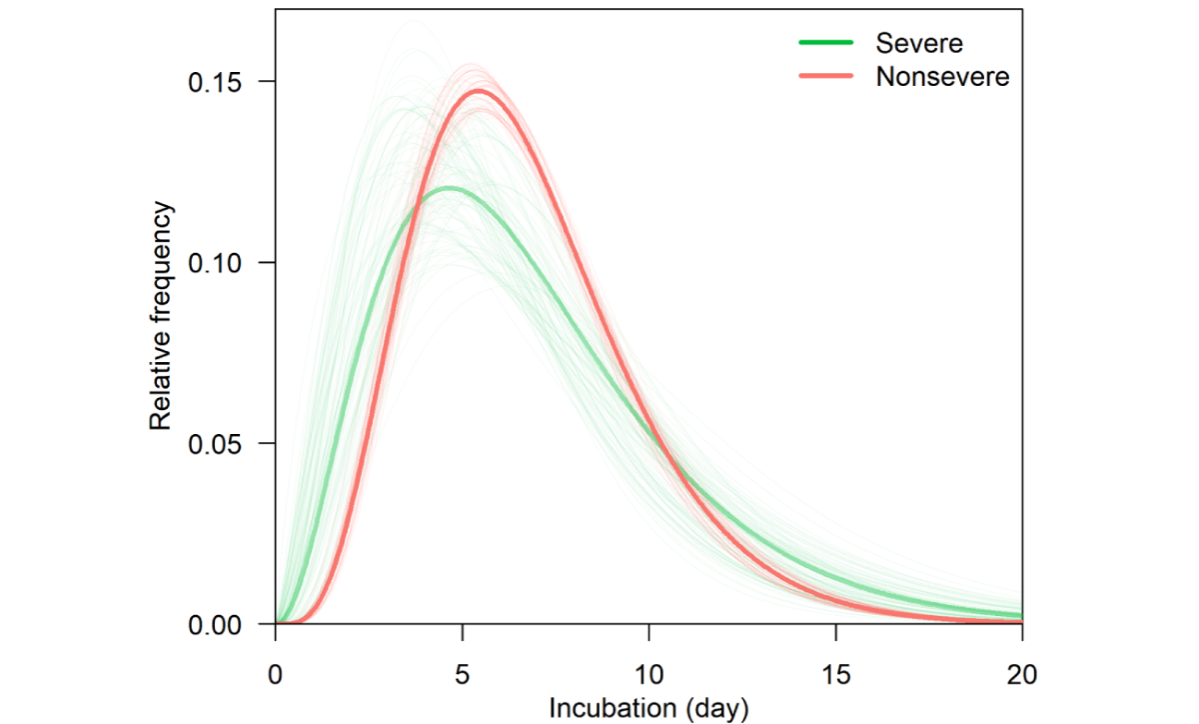
Incubation period distribution for Delta cases with severe diseases (n=31) and Delta cases with nonsevere diseases (n=322).

Figure 1 shows the exposure to the symptom onset timeline for the included cases. The estimated mean incubation period for the Delta variant was 6.64 days (95% CrI 6.27-7.00) (Figures 2 and 3). There was a trend toward longer incubation period in male cases (7.10 days, 95% CrI 6.52-7.71) compared with female cases (6.36 days, 95% CrI 5.89-6.83; Table 2).

In the age group, the mean incubation period was 6.45 days (95% CrI 5.40-7.56) for cases aged 0-18 years, 6.20 days (95% CrI 5.59-6.89) for cases aged 19-39 years, and 6.85 days (95% CrI 6.17-7.55) for those aged 40-59 years; cases aged 60-79 years had a mean incubation period of 7.02 days (95% CrI 6.34-7.76), and the shortest mean incubation period was 6.45 days (95% CrI 5.40-7.56) for those older than 80 years. The mean incubation period estimates also differed among age groups, with a shorter mean incubation period for cases aged 0-39 years and ≥80 years compared with those aged 40-79 years (Table 2).

The estimated mean incubation was shorter for critical cases (5.73 days, 95% CrI 3.83-8.11), compared with mild cases (6.41 days, 95% CrI 5.67-7.19), moderate cases (6.78 days, 95% CrI 6.34-7.25), and severe cases (6.63 days, 95% CrI 5.10-8.47). There was a trend toward longer mean incubation period for cases in Yangzhou (6.72 days, 95% CrI 6.23-7.23), compared with cases in Nanjing (6.51 days, 95% CrI 5.99-7.07; Table 2).

**Figure 4:**
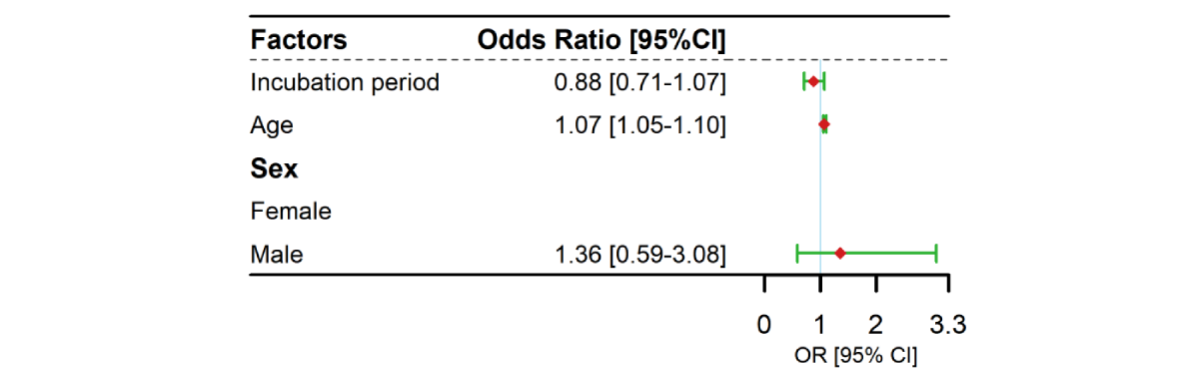
Risk factors associated with the disease severity of the Delta cases. OR: odds ratio.

The duration of incubation period had a weak and negative association with the clinical severity of COVID-19 cases infected Delta variants, with an adjusted odds ratio (OR) of 0.88 (95% CrI 0.71-1.07). After adjusting for age and sex, which implies that a 1-day increase in incubation period was associated with a 12% decrease in the probability of severe illness (Figure 4). Furthermore, age was found to be positively associated with the incubation period, with an adjusted OR of 1.07 (95% CrI 1.05-1.10).

**Table 1:** Basic characteristics of the confirmed SARS-CoV-2 Delta cases.

Characteristics			All cases (n=353)		Mild cases (n=84)		Moderate cases (n=238)		Severe cases (n=21)		Critical cases (n=10)
**Age group**											
	0-18 years	47 (13.3)		30 (35.7)		17 (7.1)		0 (0)		0 (0)	
	19-39 years	84 (23.8)		24 (28.6)		59 (24.8)		1 (4.8)		0 (0)	
	40-59 years	132 (37.4)		26 (31%)		94 (39.5)		10 (47.6)		2 (20)	
	60-79 years	83 (23.5)		4 (4.8)		64 (26.9)		10 (47.6)		5 (50)	
	≥80 years	7 (2)		0 (0)		4 (1.7)		0 (0)		3 (30)	
**Sex**											
	Male	133 (37.7)		28 (33.3)		92 (38.7)		7 (33.3)		6 (60)	
	Female	220 (62.3)		56 (66.7)		146 (61.3)		14 (66.7)		4 (40)	

**Table 2:** Estimated incubation period by sex, age groups, clinical severity, and 2 geographical regions of the Delta cases.

Characteristics			Mean (days)		Median (days)		The lower bound of 95% CrI		The upper bound of 95% CrI
Overall (n=353)			6.64		6.63		6.27		7.00
**Sex**									
	Male (n=133)	7.10		7.09		6.52		7.71	
	Female (n=220)	6.36		6.36		5.89		6.83	
**Age group**									
	0-18 years (n=47)	6.45		6.42		5.40		7.65	
	19-39 years (n=84)	6.20		6.19		5.59		6.89	
	40-59 years (n=132)	6.85		6.85		6.17		7.55	
	60-79 years (n=83)	7.02		7.02		6.34		7.76	
	≥80 years (n=7)	6.05		5.96		4.50		8.07	
**Clinical severity**									
	Mild cases (n=84)	6.41		6.41		5.67		7.19	
	Moderate cases (n=238)	6.78		6.78		6.34		7.25	
	Severe cases (n=21)	6.63		6.58		5.10		8.47	
	Critical cases (n=10)	5.73		5.66		3.83		8.11	
**Geographical region**									
	Yangzhou (n=192)	6.72		6.72		6.23		7.23	
	Nanjing (n=161)	6.51		6.51		5.99		7.07	

## Discussion

### Principal Findings

In this study, the mean incubation period of the Delta variant was estimated to be 6.64 days (95% CrI 6.27-7.00) by using the MCMC method for the interval-censored data based on uniform prior distribution *U(0,100)*. We found that a 1-day increase in the incubation period for the Delta variant was associated with a 12% decrease in the probability of severe disease after adjusting for age and sex (OR=0.88, 95% CI 0.71-1.07).

Characterizing the epidemiological features of the SARS-CoV-2 variants could provide insights into the transmission potential of COVID-19. Based on detailed contact tracing data, we estimated the incubation period of the SARS-CoV-2 Delta variant and examined the association between the incubation period and disease severity. Subgroup analysis was also conducted to investigate the difference in the incubation period distribution between age groups, sex, disease severity, and 2 geographical regions.

Our mean (6.64 days) and median (6.63 days) incubation period estimates for overall Delta cases are slightly longer than the pooled point estimates (mean: 6.3 days; median: 5.4 days) from a previous meta-analysis on the incubation period of the historical wild-type COVID-19 strains [19]. The mean incubation estimates were relatively longer than those of Grant et al [20] (6.64 days vs 4.3 days). The mean incubation estimates are also larger than previous findings from Guangdong Province, China, with a mean estimate range of 3.9 to 5.8 days [21,22]. Moreover, the mean incubation estimates were relatively longer than those by Ogata et al [23] (6.64 days vs 3.7 days). These discrepancies may be attributed to not only the biological difference between the SARS-CoV-2 strains but also the definition of the date of infection and symptom onset, as well as the estimation methodology.

The incubation period is considered as a function of the initial infectious dose, the rate of pathogen replication in the host, and intrahost defense mechanisms [24,25]. The result of mean incubation period was estimated to have a tendency for severe Delta cases to be shorter than nonsevere Delta cases (5.73 days vs 6.78 days) and for female cases to be longer than male cases (7.10 days vs 6.36 days), consistent with an earlier study using a larger sample size [15]. The multivariate logistic regression model also suggested a negative association between disease severity and incubation period even after control for age and sex. Early studies suggested that a shorter incubation period is related to a higher viral load of the initial infection, which may give rise to a more rapid pathogen replication rate that outpaces the adaptive immune system, thereby resulting in a more severe disease [26,27]. Although the biological pathway behind the incubation period and the clinical severity of COVID-19 has not been well established, a shorter incubation period may serve as an indicator of a more severe outcome for patients. Apart from that, the differences in incubation period estimates between sexes could be due to female cases exhibiting stronger innate and adaptive immune responses than male cases, which may result in a faster clearance of the in-host pathogens [28].

During the ongoing COVID-19 pandemic, estimating the distribution of the incubation period under the context of the local epidemics is essential to inform the local public health interventions such as the quarantine and isolation period [29]. With more recent data, the 95th and 97.5th percentile of the estimated distribution of the incubation period could give policy makers hints on how to adjust and improve the current control measures to effectively use the limited public health resources and at the same time to minimize the risk of permitting infectious persons into the community. Therefore, to mitigate current epidemics and prevent future outbreaks, it is crucial to obtain the incubation period estimates based on more updated epidemiological data of novel SARS-CoV-2 variants [30]. The estimates of the incubation period, across demographic and clinical features of cases, for the Delta variants added additional information to the existent evidence, which could potentially improve the policy making process.

This study has some limitations. First, the epidemiological contact tracing data were subjected to recall bias. When the confirmed cases recall their exposure window, some activities may be omitted due to unclear memory, which may lead to extra uncertainty or bias in the incubation period estimates. Second, it is possible that incubation distribution varies by vaccination status (ie, whether on is vaccinated or not), which may act as a potential confounding factor in the logistic regression model. However, because complete information on vaccination was not available, it was not included in the model. Future studies with more data could further investigate the effect of the vaccine on the incubation period of an emerging variant and explore additional factors affecting the latency of SARS-CoV-2 with more subgroup analyses. Finally, there were significantly fewer severe cases than nonsevere cases. Such imbalance may deviate the coefficient estimates.

In the future, it is necessary to further study the transmission dynamics and viral shedding, particularly for vaccinated cases with the Delta infection. Given that the current pandemic is dominated by the SARS-CoV-2 Omicron variants, it is also necessary to continue the surveillance of epidemiological characteristics of the Omicron variants.

For the novelties of this study, we adopted a state-of-the-art statistical approach to examine the association between incubation period of Delta variants and potential factors, including sex, age, and clinical severity of COVID-19 illness. The samples of incubation period observations were collected from the largest COVID-19 epidemics in eastern China seeded by Delta variants with well-traced and individual-level information of each laboratory-confirmed case. Interval censoring of incubation period observations was adjusted in the likelihood-based statistical inference framework to approach the intrinsic characteristics of incubation period. As such, the estimated associations between case characteristics and incubation period reflected evidence of the intrinsic feature of COVID-19, rather than being unauthentic due to observational or sampling bias.

### Conclusions

In conclusion, this study estimated the incubation period distribution of Delta variants according to detailed contact tracing data of COVID-19 cases in eastern China. The incubation period was found varied across sex, age, and disease severity of cases. A mild negative association between incubation period of Delta variants and clinical severity of COVID-19 was reported. These findings provided additional information on the incubation period of Delta variants and highlighted the importance of continuing surveillance and monitoring of the epidemiological characteristics of emerging SARS-CoV-2 variants as they evolve.

This study uncovered differences in incubation period between age, sex, and severe disease for patients with the Delta variant, and it will help researchers uncover key areas of the combination of incubation period with the disease severity for SARS-CoV-2 Delta variants, which many researchers have not been able to explore. Thus, a new theory on the prevention of transmission of different variants of SARS-CoV-2 may be arrived at.

## Abbreviations

CrI: credible interval
MCMC: Markov chain Monte Carlo
OR: odds ratio

## Data Availability

The processed data sets used for the analyses in this study may be available on request to the corresponding author.
